# Justice sensitivity is undergirded by separate heritable motivations to be morally principled and opportunistic

**DOI:** 10.1038/s41598-022-09253-2

**Published:** 2022-03-30

**Authors:** Nikolai Haahjem Eftedal, Thomas Haarklau Kleppestø, Nikolai Olavi Czajkowski, Jennifer Sheehy-Skeffington, Espen Røysamb, Olav Vassend, Eivind Ystrom, Lotte Thomsen

**Affiliations:** 1 PROMENTA Research Center, Department of Psychology, University of Oslo, Forskningsveien 3A, 0373 Oslo, Norway; 2 Norwegian Institute of Public Health, Oslo, Norway; 3 Department of Psychological and Behavioural Science, London School of Economics and Political Science, London, UK; 4 Department of Psychology, University of Oslo, Oslo, Norway; 5 Department of Political Science, Aarhus University, Aarhus, Denmark

**Keywords:** Genetics, Psychology

## Abstract

Injustice typically involves some people benefitting at the expense of others. An opportunist might then be selectively motivated to amend only the injustice that is harmful to them, while someone more principled would respond consistently regardless of whether they stand to gain or lose. Here, we disentangle such principled and opportunistic motives towards injustice. With a sample of 312 monozygotic- and 298 dizygotic twin pairs (N = 1220), we measured people’s propensity to perceive injustice as victims, observers, beneficiaries, and perpetrators of injustice, using the Justice Sensitivity scale. With a biometric approach to factor analysis, that provides increased stringency in inferring latent psychological traits, we find evidence for two substantially heritable factors explaining correlations between Justice Sensitivity facets. We interpret these factors as *principled justice sensitivity* (h^2^ = 0.45) leading to increased sensitivity to injustices of all categories, and *opportunistic justice sensitivity* (h^2^ = 0.69) associated with increased sensitivity to being a victim and a decreased propensity to see oneself as a perpetrator. These novel latent constructs share genetic substrate with psychological characteristics that sustain broad coordination strategies that capture the dynamic tension between honest cooperation versus dominance and defection, namely altruism, interpersonal trust, agreeableness, Social Dominance Orientation and opposition to immigration and foreign aid.

## Introduction

The question of how to ensure a truly fair system of justice may be as old as humanity itself. Having a shared standard of justice in a society can substantially improve the quality of life. But systems of rules put in place in the name of justice can also be used as a tool by those in power to gain a disproportionate share of resources and privileges. On the interpersonal level, too, rules can be enforced strategically, so that a person gains more of the benefits of those rules and incurs fewer of the costs. Accordingly, John Rawls^1^ argued that systems of justice should ideally be shaped from behind a *veil of ignorance*, where decision makers do not know their own standing in society, so that they are unable to make general rules that unfairly benefit people like themselves. Similar sentiments have been put forward by others: Adam Smith claimed justice should be decided by *impartial spectators*^2^; David Hume valued the *common point of view*^3^; and Roderick Firth’s *ideal observer* is completely neutral^4^.

In Rawls’ scenario, the corrupting influence of self-interest on justice is only mitigated through fully aligning self-interest with the shared interests of everyone. A person behind a veil of ignorance is made to weigh equally the outcomes for all members of society, because they do not know where they will end up. This then leaves open the question of whether people can ever have truly impartial and principled motivations towards justice, rather than merely selfish motivations that happen to align with the greater common good. Do people value justice for its own sake, beyond just pursuing the best deal for themselves and those they care about when negotiating a social contract?

Classical theories on the evolution of morality suggest that people indeed have such conflicting moral motivations. Moral sentiments are here thought to originate from the dynamic relation between cooperation and defection^5,6^. The principled employment of common moral rules for mutually beneficial cooperation (e.g., for reciprocity) might yield adaptive advantage in the context of a highly social species^7,8^ but is only possible to sustain insofar as cooperators are able to discriminate and deter defectors from reaping the benefits of cooperation without contributing to its costs^6^. Conversely, defectors will benefit from not being found out and punished. Evolutionary theory suggests that this will create an evolutionary arms race of adaptations, counter-adaptations, and counter-counter-adaptations for cooperative and defecting strategies regarding moral behavior—and their genetic substrates—within the species^5,9^.

In the present study, we investigate this perspective on moral motivations as it applies to the Justice Sensitivity (JS) scale^10,11^. This scale measures how readily someone perceives injustice in the world around them. Importantly, the scale has four facets, reflecting how the propensity to perceive injustice can vary depending on the perspective from which the injustice is experienced. The four perspectives are those of a *Victim*, an *Observer*, a *Beneficiary*, and a *Perpetrator*. The *Victim* facet represents sensitivity to injustice of which you are yourself a victim. *Observer* is sensitivity to injustice you observe as a bystander, without being directly involved. *Beneficiary* represents a tendency to feel guilty about passively benefiting from injustice. *Perpetrator* is the propensity to see one’s own behavior as constituting injustice towards others, and to then feel shame and guilt.

We investigate the possibility that the typical pattern of correlations between the JS facets—all substantially correlated with each other, except that victim is relatively less strongly correlated with beneficiary and perpetrator—can be explained by two separate and more general justice sensitivity factors, which we label principled and opportunistic. Our large genetically informative sample of mono- and dizygotic twin pairs allows a more comprehensive approach than has hitherto been attempted to identifying these kinds of latent traits^12^. It also enables us to explore the extents to which principled and opportunistic morality are genetically or environmentally formed. We then investigate correlations between scores on these factors with theoretically relevant measures.

### Principled justice sensitivity

To have a principled moral motivation for justice is to see justice as valuable in itself, regardless of who stands to lose or benefit from it in a given situation. David Hume^3^ thought such motivations exist, proposing that we have a moral sense which produces intuitions from a purely impartial perspective; “the common point of view”. These intuitions then compete with all our other concerns before they influence behavior. Recent work in moral psychology supports Hume’s view that we can indeed have genuine commitments to moral standards^13,14^. Relatedly, studies using economic games have shown that people can sometimes act as if “virtue is its own reward”, choosing to sacrifice their own financial gain in order to punish unjust others^15,16^. Being seen by one’s social group as impartial and committed to moral rules is valuable from a game theoretic standpoint^17^, as this increases others’ expected returns from mutually beneficial cooperation. Consistent with this, the preservation of a narrative of moral consistency to self and others could be a basic psychological need^18,19^. If people indeed experience principled moral motivations, and if they do so to varying degrees, then this should contribute to positive correlations between the four facets of the Justice Sensitivity scale. An increased concern with justice for its own sake would lead to higher sensitivity to all injustice, regardless of whether one is a victim, observer, beneficiary, or perpetrator. We label this hypothesized common factor *principled justice sensitivity* (Principled-JS).

**Figure 1:**
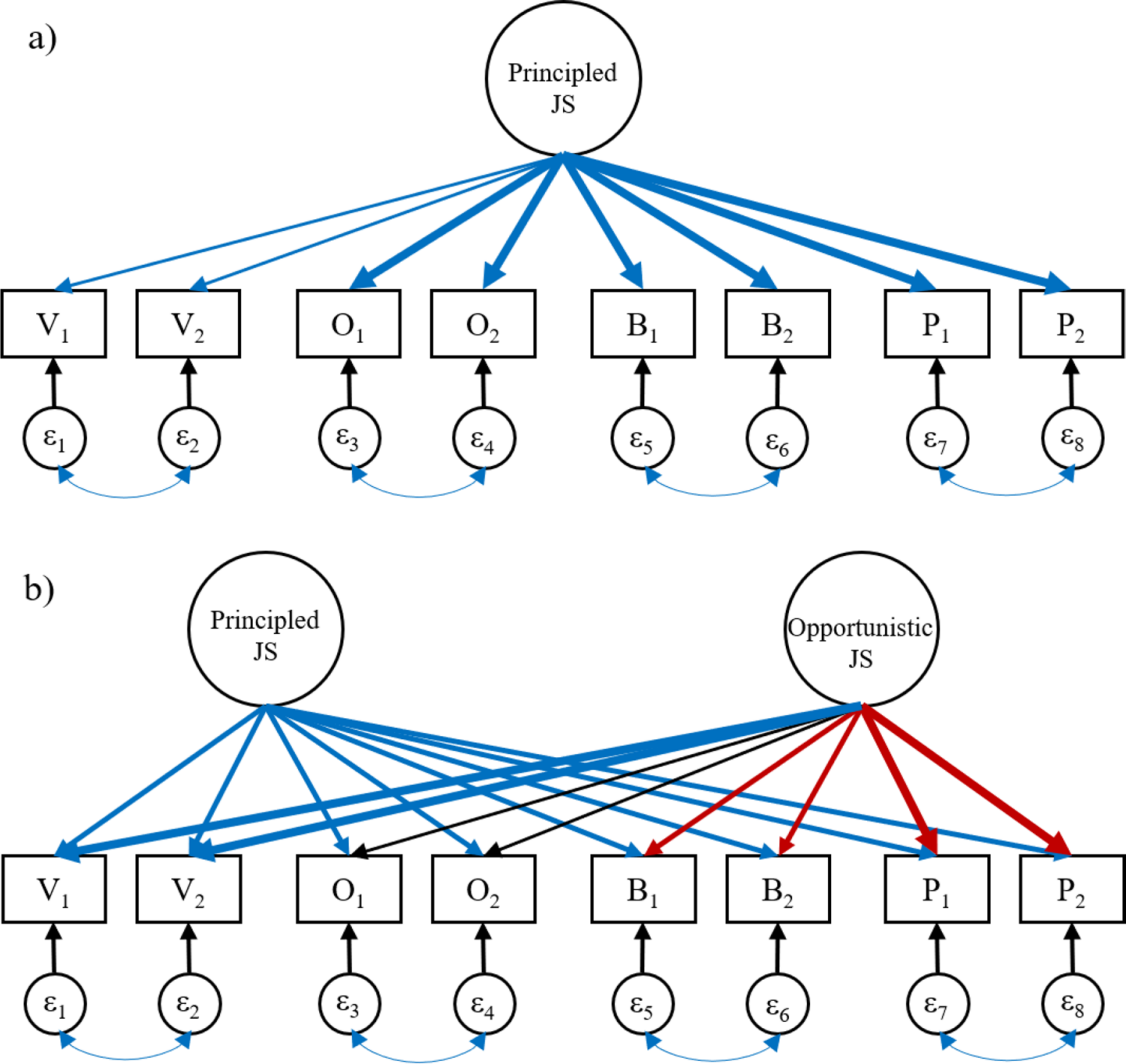
Illustrations of factor models to explain empirical patterns of correlations between Justice Sensitivity-facets. V, O, B, and P represent the JS facets of Victim, Observer, Beneficiary, and Perpetrator sensitivity, respectively, with numbers distinguishing between different items in the scale from the same facet. Line colors indicate the hypothesized valence of loadings, with blue being positive and red being negative. Line thickness indicates the strength of loadings. (**a**) Model with a Principled-JS trait influencing all facets; (**b**) Model with separate Principled-JS and Opportunistic-JS traits influencing the JS facets.

While each perspective on justice sensitivity might be influenced in the same way by such a morally principled trait, they also differ, for example, in the emotional responses they tend to elicit. Being a victim or observer of injustice typically elicits anger and indignation, while being a beneficiary or perpetrator rather leads to shame and guilt^20^. Furthermore, being a victim or perpetrator can be distinguished from being an observer or beneficiary in that the former involve a higher degree of personal involvement, leading to more intense motivations. The four-facet structure of the JS scale reflects these differences and has been supported by several studies, both for a long version of the scale with ten items per facet^10,21^, and for a short version with two items per facet^22^. Nevertheless, the empirical record suggests that the four JS facets do all tend to correlate^23^, mostly between r = 0.30 and r = 0.80, consistent with the existence of a general trait of principled justice sensitivity. While models combining pairs of JS facets have been shown to produce worse fit^21^, it might still be the case that a principled justice sensitivity exists *in addition to* specific sensitivity to being the victim, perpetrator, observer, or beneficiary of injustice. This would be represented by a single factor model (Fig. 1a) which also allows for influences that are specific to each facet of the JS scale, as represented by the correlated errors on items belonging to the same facet.

However, this principled justice sensitivity factor fails to explain an interesting empirical pattern in how justice sensitivities tend to correlate: victim sensitivity tends to correlate relatively less strongly with beneficiary and, in particular, perpetrator sensitivity, even as they all correlate substantially with observer sensitivity^21,22^. A one-factor model would only partly be able to account for this, through having weaker loadings on victim sensitivity. Rather, the pattern could suggest that an additional factor is at play, which pulls victim sensitivity in one direction and beneficiary and perpetrator sensitivity in the other (Fig. 1b).

### Morally opportunistic justice sensitivity

The idea that people may make judgments about justice in instrumental, opportunistic ways is supported by an emerging literature in moral psychology and motivated reasoning^24,25^. For instance, in judging the severity of violations of norms related to freedom of speech, people assessed restrictions of speech they supported as more blameworthy than the same restrictions imposed on speech they opposed—even while explicitly denying that speech content should play any role in such judgments^26^. Such moral selectivity, too, may be a trait which people possess to varying degrees. In the context of Justice Sensitivity, we will label this trait of being self-servingly selective about morality as *Opportunistic Justice Sensitivity* (Opportunistic-JS).

Evidence that opportunistic moral selectivity (or at least its inverse) may be an enduring personality trait is found in studies of the Honesty/Humility-dimension of the HEXACO personality inventory, tapping trustworthiness, modesty, and a lack of greed^27^, and of the Morality facet of Agreeableness in the IPIP-NEO, which reflects an aversion to using cheating and deception to get ahead^28^. Indeed, such measures have been found to be associated with responses to the JS scale: Baumert, Schlösser, and Schmitt^29^ found that the honesty/humility factor correlated negatively with victim sensitivity but positively with the sensitivity to being the beneficiary, observer, and, most substantially so, the perpetrator of injustice.

Presumably, an opportunistic selectivity in using moral rules would increase victim sensitivity and decrease beneficiary and perpetrator sensitivity. High victim sensitivity can lead to increased compensation in terms of resources and privileges, while decreased beneficiary and perpetrator sensitivity implies a decreased willingness to compensate others. Thus, opportunistic justice sensitivity maximizes material gains and minimizes losses from being sensitive to injustice, at least in the short term (until others catch on to one’s true motivations). Consistent with this proposal, victim sensitivity has been found to predict selfish behavior across several kinds of economic games^30^, and to correlate positively with Machiavellianism, neuroticism, and jealousy^10^. Furthermore, the people who are most opportunistic or Machiavellian in their use of moral rules have been found to be the least prone to feelings of shame and guilt, which are in turn the main emotions associated with sensitivity to being a perpetrator of injustice^31^. Indeed, opportunistic justice sensitivity may especially reduce sensitivity to being the perpetrator of injustice, because expectations for you to personally compensate others are stronger if you have perpetrated an injustice yourself, as opposed to simply observing or passively benefitting from injustice^32^. The possibility that opportunistic justice sensitivity might be another latent trait, in addition to principled justice sensitivity, which underlies the relationships between sensitivity to being the victim, perpetrator, observer, and beneficiary of injustice, is represented by a two-factor model (Fig. 1b). Here, all items are specified to load on both a principled and an opportunistic latent factor.

### The present research

The current study investigates the hypothesis that there are two distinct constructs underlying moral motivations towards justice, representing principled and opportunistic justice sensitivity. To do this, we first seek to identify such latent constructs, by fitting and comparing genetically-informative common and independent pathway (CP and IP) models on the Justice Sensitivity scale, while also running a standard phenotypic confirmatory factor analysis for reference. As described further in the methods section, our biometric approach to factor analysis allows more stringent inferences about latent constructs. And it also allows us to estimate proportions of variance for latent factors in our models attributable to genes versus environmental influences. Our analyses thus also bear on debates regarding the origins of moral character. Prior research suggests that situational and structural factors impact attitudes and beliefs about justice^33^. On one hand, this might suggest that shared-environmental factors among twins, such as socioeconomic status and parenting, will play a more prominent role in explaining variability in such moral justice concerns than is typically found for other psychological traits. Shared-environmental factors like these typically account for less than 15% of variance in psychological traits, while genes typically account for between 30 and 60% of variability^34^. On the other hand, several other pro- and anti-social traits that are relevant for cooperative versus opportunistic morality have been found to be heritable, with little or no contribution from the shared environment, including Machiavellianism^35^, psychopathy^36^, dispositional empathy^37^, altruism^38^ and trust^39^. This suggests that moral sensitivity to injustice may also be more strongly influenced by genes than the shared environment, as has recently been found in a Chinese sample^40^.

Finally, biometric modeling can shed light on justice sensitivity’s relationship to, and distinctness from, related psychological traits. One pressing concern is whether any such heritable genetic substrate for justice sensitivity is distinct from, or simply reduces to, Big Five personality traits (e.g., agreeableness)^41^ that are phenotypically correlated with justice sensitivity^21^, and are themselves heritable^34^. This addresses the general theoretical question of whether moral character and values are distinct from the temperament and action tendencies which personality inventories typically tap^42^. A further critical question we investigate is whether any such distinct, heritable strategies for principled versus opportunistic justice sensitivity share common genetic substrates with other psychological traits that serve to sustain cooperation for the greater common good—such as altruism^38,43^ and trust^39^—versus serve to maximize adaptive fitness gains over others, such as social dominance and the monopolization of territory and resources^44^.

## Materials and methods

### Sample

The twins in our sample were recruited from the Norwegian Twin Registry (NTR), which consists of several cohorts of twins. Our cohort consisted only of same-sex twins, born between 1945 and 1960. The questionnaire was filled out in 2016. The mean age was 65.16 (SD = 4.49). For complete pairs with valid scores on our scales of interest, our sample consisted of 121 male monozygotic twin pairs, 124 male dizygotic pairs, 191 female monozygotic pairs, and 174 female dizygotic pairs. This yielded a total of 610 twin pairs, or 1220 subjects. Zygosity was determined by a questionnaire shown to correctly classify > 97% of twins^45^. Our study was approved by the Regional Committee for Medical and Health Research Ethics of South-East Norway, and all our methods comply with their guidelines and regulations, and with the Declaration of Helsinki. Informed consent was obtained from all research participants.

### Measures

Among the measures in the questionnaire, we include the following measures in our analyses:

#### Justice sensitivity (JS)

We used the short version of the Justice Sensitivity scale^22^, which has a total of eight items: two items each for *Victim*, *Observer*, *Beneficiary*, and *Perpetrator* sensitivity. For example, the item “It makes me angry when others are undeservingly better off than me” is part of the Victim facet. Agreement to each item was indicated using a 7-point Likert scale (1 = strongly disagree, to 7 = strongly agree). Cronbach’s α for the scale as a whole was 0.77.

#### Social dominance orientation (SDO)

Social dominance orientation was measured with the SDO_6_^46^, a 16-item scale that reflects one’s preferences for the unequal distribution of power and resources between groups in society (α = 0.85).

#### Interpersonal trust

Our measure of interpersonal trust is from the European Social Survey^47^. It consists of three items indexing whether respondents tend to see other people as generally trustworthy (α = 0.82).

#### Altruism

The Self-Report Altruism scale measures the self-reported frequency of a set of five selfless behaviors, such as donating blood or money^48^ (α = 0.48).

#### The big five inventory (BFI)

The BFI is a well-established taxonomy of personality, with the five dimensions of *Openness to experience* (α = 0.78), *Conscientiousness* (α = 0.73), *Extraversion* (α = 0.80), *Agreeableness* (α = 0.72), and *Neuroticism* (α = 0.83)*.* Our survey included the BFI-44^41^, which is a shortened 44-item version of the full BFI scale.

#### Resource monopolization: attitudes towards immigration and foreign aid

Our survey included four questions about attitudes towards immigration policy and foreign aid. Responses to these questions correlated highly, and so we combined them into a single scale (α = 0.77) where high scores correspond to less support for immigration and foreign aid, thus reflecting the extent to which one advocates restricting national resources to Norwegians.

### Analyses

#### Twin models

The classical twin design allows partitioning the variation of a trait into three components: *A*, *C*, and *E*. A is additive genetic influences, C is shared-environmental influences, and E is unique-environmental influences. Genetic effects cause monozygotic (MZ) twins to be more similar to each other than dizygotic (DZ) twins are, shared-environmental effects are everything that makes twins similar to each other except genetics, and unique-environmental effects are everything that makes monozygotic twins different from each other. Thus, in addition to individual experiences, the unique environment also contains measurement error as well as the intrinsic molecular stochasticity in the process of building a phenotype from a genotype^49^. If shared-environmental influences are the only reason twins correlate on a phenotype, then MZ and DZ pairs should correlate equally. The more strongly MZ pairs are correlated compared to DZ pairs, the more of the variance in that phenotype is then attributed to genes and the less is attributed to the shared environment. We used structural equation modeling to model the covariances of the responses of twins in terms of additive genetic effects, shared-environmental effects, and unique-environmental effects^50^. These models were fitted by full-information maximum likelihood analysis using OpenMx^51^. As we focus here on general aspects of Justice Sensitivity rather than sex differences, sex was regressed out of the justice sensitivity scores, and standardized residuals were used in subsequent twin analyses^52^.

#### Common and independent pathway biometric models

**Figure 2:**
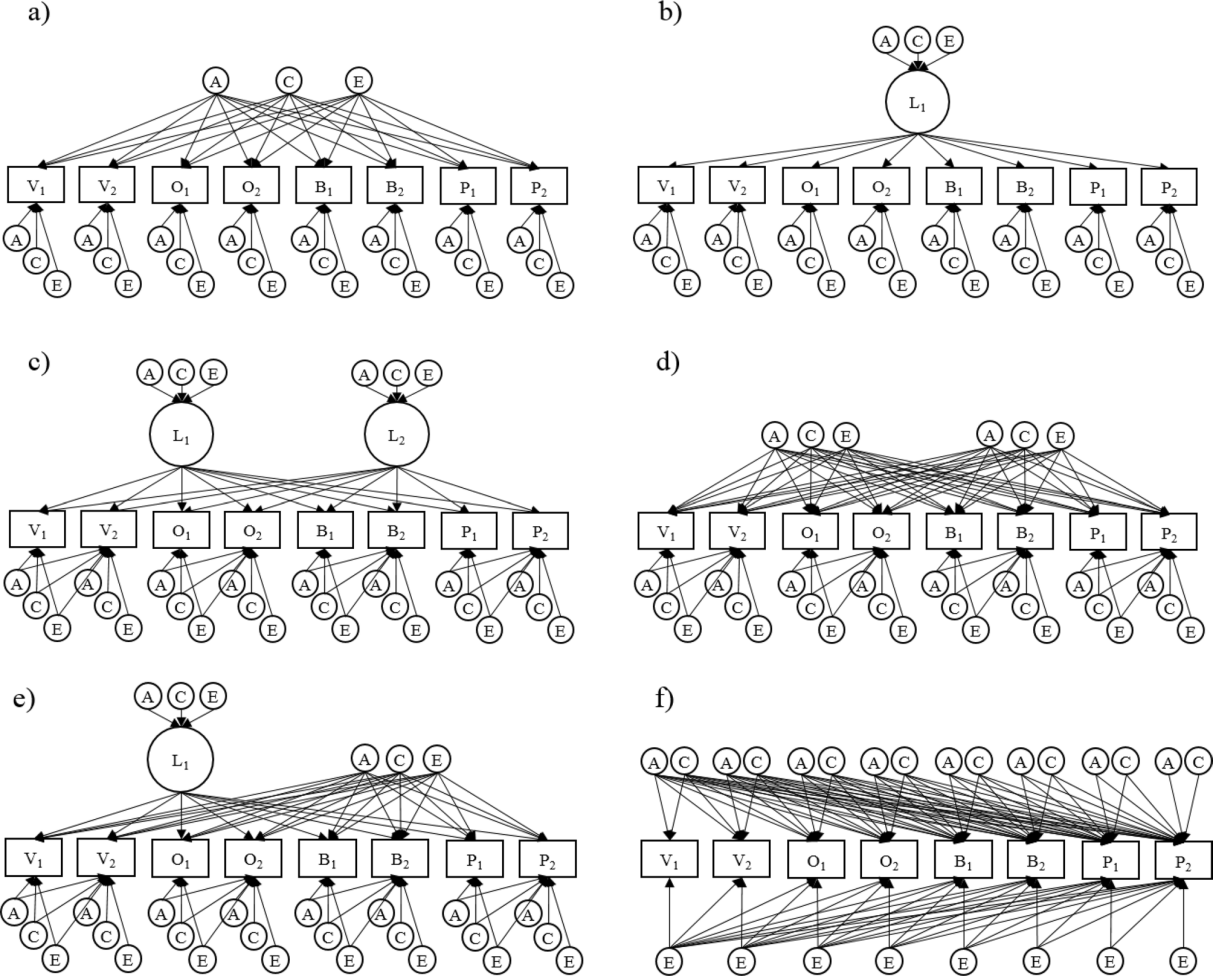
Illustrations of multivariate twin models. (**a**) 1-factor IP model; (**b**) 1-factor CP model; (**c**) 2-factor CP model allowing items from the same JS facet to share residual variance (the models in d and e also allow for this); (**d**) 2-factor IP model (**e**) hybrid model with one CP factor and one IP factor; (**f**) Cholesky model, working as our baseline for comparisons.

As an elaboration on standard factor analytic models with latent factors, twin data allow fitting models with separate A, C, and E components. These are called Independent Pathway models (“IP model”; see Fig. 2a), wherein a set of separate A, C, and E components constitute a single “IP factor”. Such a model then partitions the sources of covariances into A, C and E.

If, in fact, the true reason for the covariances between observations is the existence of a single latent trait, then the patterns of loadings (i.e., the relative magnitudes of loadings) from the A, C, and E components should be equal. After all, in this situation, genetic and environmental inputs could only affect covariances through first affecting the latent trait, which has a specific pattern of loadings on the observed variables. When adding such a latent trait to the model, and specifying that all influences on covariances of items must occur through first affecting this trait, the model becomes a Common Pathway model (“CP model”; Fig. 2b). A CP model is then equivalent to an IP model where loading patterns from the A, C, and E components are constrained to be equal. Adding this constraint reduces the number of estimated parameters, and thus will lead to improvements in parsimony-corrected fit indices if covariances are indeed explained by a single latent variable. Conversely, the more divergence there is from this situation of a single latent trait, the more genetic and environmental loading patterns will tend to differ (for more details, see e.g., Franic et al.^12^). IP models would then become superior to CP models. If an IP model fits better than a CP model in a situation where phenotypic models suggest a one-factor solution, this suggests that the real reasons for the covariances are in fact more complex than just the single factor from the phenotypic model.

For cases with more than one latent factor, such as in our hypothesized scenario with both Principled-JS and Opportunistic-JS traits, it is possible to compare CP and IP models with more than one factor. A CP model would then correspond to there being several *sets* of genetic and environmental factors with the same patterns of loadings: one set for each latent trait (Fig. 2c). IP models are equal to these models, except that they again lack the constraint of equal loading patterns; for each latent trait, separate A, C, and E factors are estimated (Fig. 2d). In a situation with two factors, it is possible that one of them is a genuine latent trait, while the other one is not. Such a situation can be represented by a CP-IP hybrid model, with one CP factor constrained to have equal loading patterns, and one IP-factor without this constraint. (Fig. 2e). We compare models with either one, two, or three factors, in CP, IP, and CP-IP hybrid versions. Models with two or less factors represent the existence of at least one of the hypothesized Principled-JS and Opportunistic-JS traits. Models with three factors allow for the existence of additional, unforeseen traits also contributing to correlations between JS facets. IP models with multiple factors can only be fitted with orthogonal factor rotations, so we only investigate orthogonal factor rotations for CP models as well, for cleaner comparisons.

Variance for each item that is unexplained by the latent factors is divided into separate A, C, and E components. To account for how items belonging to the same JS facet share variance beyond that explained by latent factors, our models allow the variance components for the first item in a pair to also load on the second item in the pair.

As a baseline for comparisons, we use a model with a standard Cholesky decomposition of the variance shared between items (Fig. 2f). This is a type of model that simply allows all the observed variables to have correlated genetic and/or environmental influences, without specifying any broader latent factors as the sources of these correlations. It is a highly flexible model that can accommodate most kinds of explanations for why the JS facets correlate, including situations without a simple structure with a small number of factors.

In addition to the full ACE versions of the models, we estimate AE and CE versions where C or A components are removed. An IP factor in an AE model then consists of just an A factor and an E factor, and likewise for CE models. Since our approach is still quite far from exploring the full space of possible models, we also investigate if our best-fitting model can be further improved by removing or adding single A, C, or E components.

Since the models we are comparing are not nested within the baseline Cholesky model, we use Akaike’s Information Criterion^53^ (AIC) to adjudicate between them.

For completeness, we also conduct a traditional phenotypic Confirmatory Factor Analysis on our data, assessing for the same set of two latent traits.

## Results

### Descriptives

Descriptive statistics for all measures and twin correlations are given in Table 1.

**Table 1:** Descriptive statistics and twin correlations.

JS facet	r	Mean (SD)	Twin correlations (95% CI)	
			MZ	DZ
Victim	0.70	2.69 (1.34)	0.35 (0.28, 0.41)	0.17 (0.10, 0.24)
Observer	0.71	4.32 (1.42)	0.29 (0.22, 0.36)	0.12 (0.04, 0.19)
Beneficiary	0.80	3.00 (1.50)	0.30 (0.23, 0.37)	0.10 (0.02, 0.17)
Perpetrator	0.81	5.25 (1.81)	0.26 (0.19, 0.33)	0.16 (0.08, 0.24)

r = correlation between the two items for each facet.

Table 2 shows the correlations between JS facets. Here, it is noteworthy that the correlation between Victim and Perpetrator is even lower than in other contexts, at r = 0.08. Furthermore, the correlation between Beneficiary and Perpetrator, which is typically the highest among all JS facet correlations, is also remarkably low in our sample, at r = 0.29 (compared to e.g., Baumert et al.^22^, who reported correlations at 0.72 and 0.69).

**Table 2:** Phenotypic correlation matrix for the four JS facets (with 95% CIs).

	JS-victim	JS-observer	JS-beneficiary
JS-observer	0.34 (0.30, 0.38)		
JS-beneficiary	0.38 (0.34, 0.42)	0.47 (0.44, 0.50)	
JS-perpetrator	0.08 (0.03, 0.12)	0.32 (0.27, 0.36)	0.29 (0.25, 0.33)

All correlations are significant at p < 0.01.

### Confirmatory factor analysis

Exploring whether correlations between JS facets are best described by one or two latent factors first in a standard CFA framework, we fitted the two models shown in Fig. 1. All models were fitted with standard errors that were correlated for each twin pair, to account for non-independencies of observations. We also fitted these models without the correlations between residuals from items belonging to the same facet. Model fit deteriorated substantially when these correlations were removed. See Table 3 for statistics for the different models.

**Table 3:** Comparisons for phenotypic CFA.

Model	EP	AIC	RMSEA	CFI
1-factor no-cor	16	40,991.48	0.198	0.58
1-factor cor	20	39,020.09	0.062	0.97
2-factor no-cor	24	39,850.77	0.160	0.80
**2-factor cor**	**28**	**38,884.62**	**0.033**	**0.99**

“cor” and “no-cor” signify whether a model is fitted with- or without correlations between residuals for items belonging to the same facet, respectively; *EP* number of estimated parameters, *AIC* Akaike’s Information Criterion, *RMSEA* Root Mean Squared Error of Approximation, *CFI* Confirmatory Fit Index. Best fitting model indicated in bold.

The model with two latent factors (Fig. 1b) provided better fit than the model with only one such factor (Fig. 1a). RMSEA for this model was 0.033 and CFI was 0.99, meaning that it fit the data adequately.

**Figure 3:**
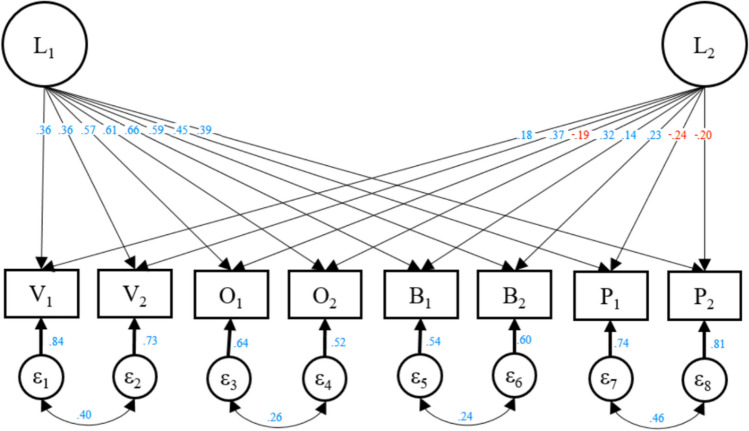
Best fitting model from phenotypic CFA. Illustration of our best fitting model from phenotypic CFA. Blue numbers are positive and red numbers are negative.

The full model with parameter values is shown in Fig. 3. The first factor aligns with our proposed Principled-JS trait, with substantial positive loadings on all JS items. The second factor aligns with Opportunistic-JS to some extent, in that it has positive loadings on JS-Victim and negative loadings on JS-Perpetrator. Less expectedly, loadings on JS-Beneficiary are positive, and loadings on the second item in each pair from the same JS facets are consistently more positive than for the first item in the pair (particularly so for JS-Observer).

### Biometric factor models

We compare CP, IP, and CP-IP hybrid models with one, two, or three factors (see Fig. 2 for illustrations). As a baseline model for comparisons, we use a full Cholesky decomposition of variance. The fit statistics for these models are detailed in Table 4. Pure AE models (with all C’s taken out) were consistently superior to models removing only a subset of the C’s. So, for clarity, we present statistics only for pure AE models here (except the baseline model, “Cholesky ACE”). Similarly, allowing items from the same JS facet to share variance beyond that accounted for by common factors was also consistently preferable, so all models in the table have this property as well.

**Table 4:** Model comparisons for multivariate twin models.

Model	EP	− 2LL	df	Δ − 2LL	Δ df	AIC	p
**Initial model comparisons**							
Cholesky ACE	116	55,053.12	15,353	NA	NA	24,347.12	NA
Cholesky AE	80	55,062.06	15,389	8.940	36	24,284.06	0.999
CP1	42	55,276.22	15,428	223.100	75	24,420.22	< 0.001
CP2	52	55,127.60	15,419	74.475	66	24,289.60	0.222
CP3	62	55,083.79	15,410	30.668	57	24,263.79	0.998
IP1	48	55,190.09	15,421	136.962	68	24,348.09	< 0.001
IP2	64	55,081.24	15,405	28.117	52	24,271.24	0.997
IP3	80	55,066.91	15,389	13.788	36	24,288.91	0.999
**CP1_IP1**	**58**	**55,085.92**	**15,414**	**32.794**	**61**	**24,261.92**	**0.998**
CP1_IP2	74	55,067.49	15,400	14.364	47	24,267.49	0.999
CP2_IP1	68	55,074.08	15,405	20.961	52	24,264.08	0.999
**Adjustments of best model**							
**CP2_E1**	**60**	**55,083.79**	**15,412**	**30.668**	**59**	**24,259.79**	**0.999**
CP2_A1	60	55,276.22	15,428	223.100	75	24,420.22	< 0.001
CP1_IP1_E1	62	55,083.79	15,410	30.668	57	24,263.79	0.998
CP1_IP1_A1	52	55,127.60	15,419	74.475	66	24,289.60	0.222
CP2_E1_Apath	61	55,083.79	15,411	30.668	58	24,261.79	0.999

*EP* number of estimated parameters, *LL* LogLikelihood, *df* degrees of freedom, *Δ* difference as compared to base model, *AIC* Akaike’s Information Criterion, *p* p-values from test of difference from base model. Best fitting model indicated in bold. CP and IP stand for common and independent pathway respectively. The numbers after CP and IP indicate the number of factors of that kind in the model. E1 signifies the addition of an extra E factor to a model. Apath represents a path from the A-component of a CP-factor onto the other CP-factor. All models in the table except the Cholesky ACE are pure AE models, with no C components.

Our best-fitting model from this initial comparison was a 2-factor CP-IP hybrid model (CP1_IP1). As large parts of the space of possible models are still unexplored, we tested adjustments to this model involving the addition of a single variance component. This new component either comes in the form of an additional pure A or E factor, or it can be added to the existing A or E factors making up the IP factor in the model so that they become CP factors. (Models involving the subtraction of variance components, and models involving C components, were also tested).

The best-fitting model then becomes a 2-factor CP model with an additional pure E factor (CP2_E1), created by adding an E component to the pure A factor in the CP1_IP1. This model can be seen as a compromise between the two best-fitting models in the initial step: CP1_IP1 and CP3. As the third factor in the CP3 naturally came out as 100% environmental, these two models are almost exactly alike, except that one of the factors in CP1_IP1 is constrained to be 100% genetic (The corresponding CP-factor in CP3 is 69% genetic). CP2_E1 achieves better fit than CP3 only due to having fewer estimated parameters, as the third factor is now constrained to be purely environmental. No further improvements in fit could be achieved from adding or subtracting components for this CP2_E1 model. Importantly, this includes the addition of a path from the A-component of the first CP-factor onto the second CP-factor (CP2_E1_Apath). Such a path can be seen to represent the possibility that the two CP-factors are genetically correlated. However, the results from fitting this model suggest that the two factors are remarkably independent even when they are not constrained to be, as the parameter estimate for the added A-path came out to − 0.09, and was not significant.

**Figure 4:**
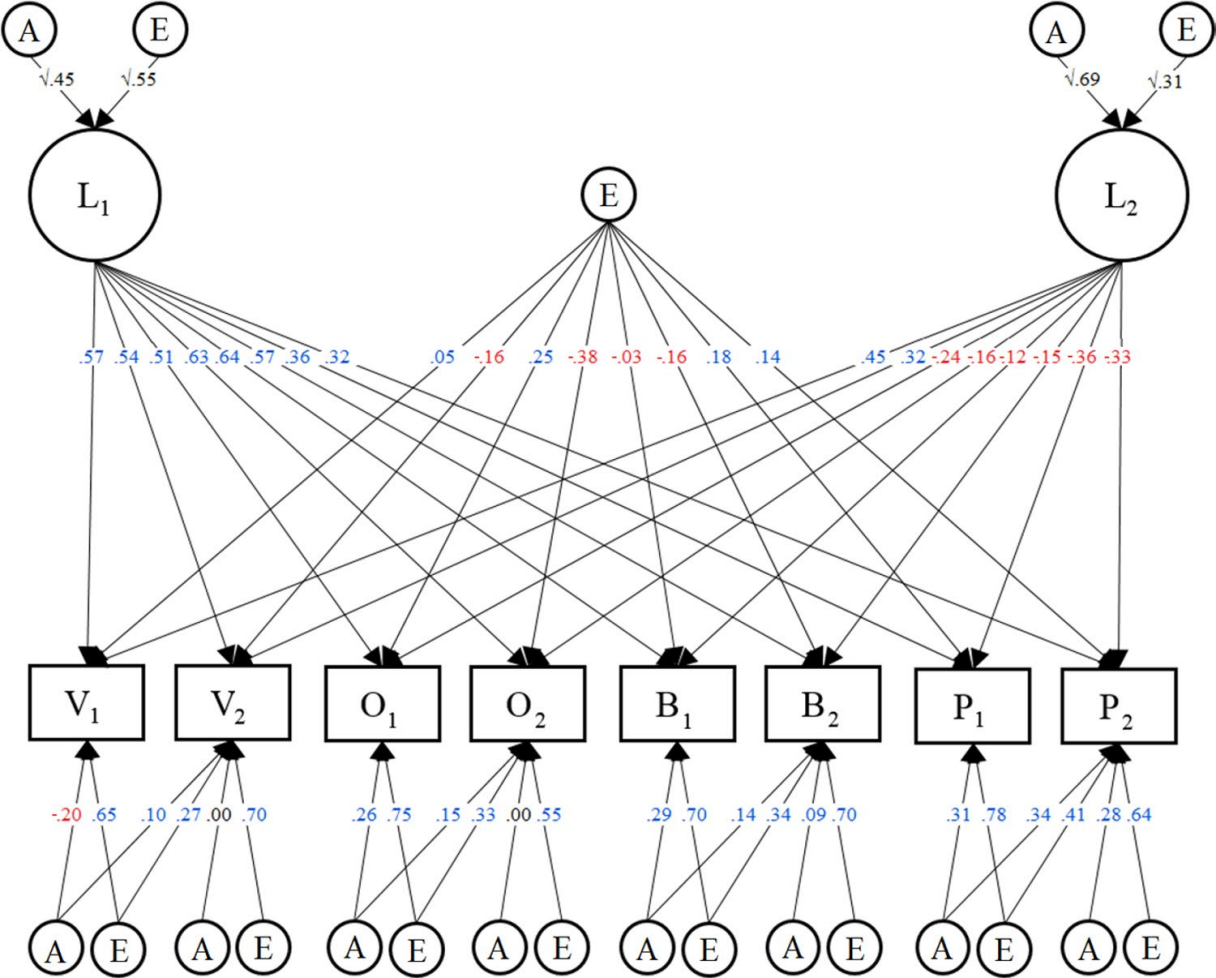
Best fitting biometric factor model. Illustration of our best fitting biometric factor model. Blue numbers are positive and red numbers are negative.

Parameter values for CP2_E1 remained similar to those for CP3 and CP1_IP1. The first CP factor corresponded well with the hypothesized Principled Justice Sensitivity (Principled-JS) construct, having significant positive loadings on all the JS items. The second CP factor corresponded to Opportunistic Justice Sensitivity (Opportunistic-JS), with positive loadings on Victim items and negative loadings on the rest, with loadings on Victim and Perpetrator items being of larger magnitude than those for Observer and Beneficiary items. Both of these factors were substantially heritable, with heritability estimates at 45% for the Principled-JS factor and 69% for the Opportunistic-JS factor. The purely environmental factor had largely alternating positive and negative loadings of modest magnitude. Plausibly, this could reflect correlations in measurement errors from how pairs of items are formulated. See Fig. 4 for an illustration of the model, with all its parameter values. In combination, the three latent factors in the top part of this model explain on average 40% of the variance in each JS item, ranging from 57% (for item O_2_) to 23% (for item P_2_).

### Correlations of factor scores with cooperative versus dominating strategies and with Big Five personality traits

We calculated factor scores for the Principled-JS and Opportunistic-JS factors in the best-fitting twin model, and correlated these with cooperative (indexed here with Altruism and Interpersonal Trust) and dominating (indexed here with SDO and opposition to immigrants and foreign aid) strategies, as well as with Big Five personality traits. Owing in part to our large dataset, most of our correlations were significant, with p-values far below 0.05, even though phenotypic correlations were generally modest in size (See Table 5).

**Table 5:** Correlations between factor scores and relevant traits.

	Principled-JS			Opportunistic-JS		
	rP	rA	rE	rP	rA	rE
SDO	**− 0.10**	**− 0.13**	**− **0.08	**0.27**	**0.40**	**0.22**
Immi-aid	**− 0.13**	**− 0.25**	**− **0.05	**0.25**	**0.60**	**− **0.01
B5O	0.02	0.06	**− **0.01	**− 0.16**	**− 0.28**	**− **0.07
B5C	**− 0.13**	**− 0.26**	**− **0.06	**− **0.03	**− 0.10**	**− **0.00
B5E	**− **0.08	**− 0.18**	**− **0.01	**− **0.05	**− 0.17**	0.05
B5A	**− 0.12**	**− 0.14**	**− 0.10**	**− 0.17**	**− 0.19**	**− 0.16**
B5N	**0.17**	**0.35**	0.07	0.08	**0.15**	0.03
Altruism	0.05	0.05	0.06	**− 0.18**	**− 0.46**	**− **0.01
Trust	**− **0.02	**− 0.10**	0.02	**− 0.20**	**− 0.43**	**− **0.09

*Principled-JS* factor scores from the factor with significant positive loadings on all 8 JS items, *Opportunistic-JS* factor scores from the factor with positive loadings on the two Victim sensitivity items and negative loadings on the remaining 6 items, *rP* Phenotypic correlation, *rA* Additive genetic correlation, *rE* unique-environmental correlation, *SDO* Social Dominance Orientation, *Immi-aid* opposition to immigration and foreign aid, *B5O* Big Five Openness to Experience, *B5C* Big Five Conscientiousness, *B5E* Big Five Extraversion, *B5A* Big Five Agreeableness, *B5N* Big Five Neuroticism, *Altruism* Frequency of various altruistic behaviors, *Trust* Interpersonal trust. Bold font is used for numbers > |0.10|, to highlight practical significance. All correlations larger than |0.07| are statistically significant at p < 0.01.

As predicted, at the phenotypic level the Opportunistic-JS factor had positive correlations with Social Dominance Orientation and with opposition to immigration and foreign aid, but negative correlations with Altruism, and Interpersonal Trust. It also had negative correlations with Agreeableness and Openness to Experience from the Big Five.

In contrast, and as predicted, the Principled-JS factor correlated negatively with Social Dominance Orientation and opposition to immigration and foreign aid, but at the phenotypic level it was surprisingly unrelated to Altruism and Interpersonal Trust. Principled-JS was also negatively correlated with Agreeableness and Conscientiousness from the Big Five. Both Principled and Opportunistic-JS were negatively correlated with Big Five Neuroticism. As mentioned in the methods section, the factors in our model were specified to be orthogonal, so they do not correlate with each other (our model allowing them to correlate genetically, “CP2_E1_Apath”, had worse fit and produced a weak negative correlation).

Table 5 also contains genetic and environmental correlations (rA and rE, respectively). These describe the degree of overlap in genetic and environmental influences on the pairs of traits. As our genetic correlations are generally higher than the corresponding environmental correlations, the relationships between our latent factors and the other variables seem to be largely due to common genetic influences on both traits, more so than common environmental influences.

## Discussion

The tension between selfless and self-serving motivations towards injustice has long been a focus of both moral philosophy and evolutionary psychology. The present study sought to disentangle these motivations, so that their bio- and psychometric properties can be studied separately. Our results suggest (a) that responses to injustice indeed can be shaped by both morally principled and self-serving, opportunistic motivations, (b) that these motives are reflected in latent traits that are genetically grounded, and (c) that they are genetically associated with other personality and attitudinal measures reflecting broad strategies for altruistic, trusting cooperation versus dominance and resource monopolization (Cf. also^44^). The present results thus demonstrate that sensitivities to being the victim, perpetrator, observer and beneficiary of injustice are undergirded by broad, genetically heritable cooperative versus dominating strategies, which may both operate (and conflict) within the same person.

We used genetically informative data to investigate the existence of latent dispositions underlying correlations between facets on the Justice Sensitivity scale. In doing this, we avoid the pitfall of factor analyses based purely on phenotypic correlations, which can sometimes support inferring latent psychological traits that do not in fact exist^12^. The use of a large twin dataset enabled the construction and comparison of a series of biometric factor models to identify latent factors. We followed the logic that if correlations between facets of a scale are actually mediated by latent psychological traits, then when a factor is split up into separate genetic and environmental components, the patterns of loadings from these components should be highly similar, since they both affect covariances only through their effects on the same latent trait. This prediction is tested through comparing Common and Independent Pathway (CP and IP) models on the Justice Sensitivity (JS) scale, with residuals from JS items belonging to the same facet being allowed to correlate.

It should be noted that the pattern of correlations between JS facets in our sample differs somewhat from what has been observed previously^21,22^. In particular, the correlations of JS Perpetrator with both JS Victim and JS Beneficiary are lower than what is commonly observed, at r = 0.08 and r = 0.29, respectively (as compared to e.g., Baumert et al.^22^, who found correlations of r = 0.22 and 0.30 between Perpetrator and Victim, and correlations of r = 0.72 and 0.69 between Perpetrator and Beneficiary). We are not able explain these differences, beyond pointing out that our sample differs from previous samples both in terms of nationality (Norwegian vs German) and age (M = 65 in our sample, as compared to e.g., M = 47 in Baumert et al.).

We found that the best fitting model had two CP factors and an additional purely environmental factor. One of the CP factors corresponded well with the notion of principled justice sensitivity (Principled-JS), as it had substantial positive loadings on all the JS items, implying that the previously observed association between JS facets is driven by an underlying sensitivity to injustice regardless of the perspective from which it is perceived.

The other CP factor quite strongly resembled the predicted trait of opportunistic justice sensitivity (Opportunistic-JS). Loadings on JS-Victim were substantial and positive, while all other loadings—particularly those for JS-Perpetrator—were negative. This corresponds to a selective sensitivity to injustice that harms one’s interests—a form of moral opportunism. The existence of both principled and opportunistic JS traits explains how, in prior literature, JS-Victim is generally more weakly related to the other JS facets than they are to each other, both genetically^40^ and phenotypically^21^: It results from JS-Victim and the other facets being pulled in the same direction by Principled-JS, but then also (less strongly) in opposite directions by Opportunistic-JS.

The use of a large twin sample also enabled us to examine heritability in the two fundamental justice sensitivity traits. We found that the best-fitting models for our data were generally AE models, in which all shared-environmental components were taken out. This suggests that environmental influences that serve to make twins in a pair more similar to each other, such as family environments or socio-economic status in childhood, play little role in explaining variability in principled sensitivity to injustice or in moral opportunism in our sample.

We found that 45% of the variance in Principled-JS was attributable to genes, with the remaining variance being explained by non-shared-environmental influences. This heritability estimate is on a par with those generally found in the domain of personality generally^34^, and also justice sensitivity specifically^40^. The Opportunistic-JS factor was even more heritable than the Principled-JS factor, with 69% of its variance observed to be genetic in origin. It should be noted that whenever there are gene-environment correlations, such that genes cause individuals to elicit and/or actively seek out certain environmental influences on a trait, these influences will be interpreted by twin study analyses to be genetic, in line with the notion of an *extended genotype*^54^.

Finally, a third, purely non-shared-environmental, factor in our final model had generally weaker loadings than the other two factors, and these were consistently more positive for the first than the second item in each pair of items from the same JS facet. A plausible interpretation of this factor is thus that it represents correlated measurement errors, related to how pairs of items from the same facet are formulated. The first items from all pairs are formulated quite similarly across the different justice perspectives, just with differences in a few words, and the same is true for all the second items in each pair.

This possibility is consistent with comparisons between the best-fitting biometric model and the results of our phenotypic Confirmatory Factor Analysis on the same data. Here it could be seen that whereas the Principled-JS factor is present in both models, the Opportunistic-JS factor and the added E factor in the twin model are combined into a single factor in the best fitting phenotypic CFA model. Our study thus highlights that, generally, the estimation of separate genetic and environmental factors is helpful in distinguishing methodological artifacts from real psychological traits. By definition, artifacts will tend to not be influenced by genetics or family environments. Real psychological traits, in contrast, will consistently be heritable to some extent^55^. The biometric model results, in addition to the high heritability estimates for both morally principled and opportunistic justice sensitivity, thus speak against the possibility that these factors are methodological artifacts.

All models with CP and/or IP factors also include estimates of genetic and environmental influences specific to each item beyond what is accounted for by these broader factors. For all our models, fit was improved by allowing correlations between these influences for items belonging to the same specific facets of sensitivity to being the Observer, Perpetrator, Victim and Beneficiary of injustice. This is consistent with prior work on the Justice Sensitivity scale, indicating that the current division of items into these four separate facets is meaningful. The genetic components of the variance in scores left unexplained by the general factors were mostly shared between items from the same facet, while the non-genetic components of this variance were largely item specific.

### Principled and opportunistic justice sensitivity relate to broad strategies for cooperation and dominance

If our two justice sensitivity factors do in fact reflect morally principled and opportunistic strategies, then they should relate to other psychological characteristics that sustain broad coordination strategies capturing the dynamic tension between honest cooperation and defection^6,56^. Consistent with this, we found that greater opportunistic justice sensitivity is phenotypically, and substantially genetically, related to reduced altruism and interpersonal trust and increased motives for social dominance and monopolizing territory and resources (to the extent that this is reflected in one’s opposition to immigration and foreign aid). In contrast, morally principled justice sensitivity was negatively, but weakly, related to dominance and resource monopolization, but was largely unrelated to altruism and trust.

We found that principled and opportunistic justice sensitivity were only weakly related to Big Five Personality traits, indicating that they reflect moral personality (or ‘character’) traits that are distinguishable from standard personality constructs in significant ways. This addresses a concern about current personality theories, which is that they do not fully capture how people can vary in moral character^42^. While human behavior is often guided by values and moral norms, personality inventories tend mostly to index mere action tendencies and temperaments, which lack this normative component^57^.

While the two components of justice sensitivity are distinct from personality and social attitudes, we nevertheless found evidence that they have a shared genetic grounding. Generally, we found that genetic correlations were stronger than phenotypic correlations, and that environmental correlations were weak. This indicates that where the two forms of justice sensitivity do overlap with other traits and preferences, they do so because of shared genetic influences on both variables. This corresponds to Cheverud’s conjecture^58^ that genetic correlations are a representation of the relationship between variables when this is not watered down by measurement errors the way phenotypic correlations can be.

Previous research has reported negative correlations of victim sensitivity with facets of agreeableness, trust, and honesty/humility, and positive correlations with neuroticism, jealousy and narcissistic rivalry^21^. Thus, high victim sensitivity has been variously interpreted as antagonistic self-protection^59^, fear of being exploited^60^, and generally as reflecting an antisocial, uncooperative disposition^23^. The present study suggests that these descriptions are perhaps better applied to the broader Opportunistic-JS factor, rather than just to JS-Victim.

One surprising result was that of a weakly *negative* correlation between Principled-JS and agreeableness. A possible interpretation of this could be that the desire to uphold justice that comes with having a high score on Principled-JS can sometimes come into conflict with the desire to maintain social harmony associated with agreeableness. Interestingly, the personality facet most strongly (genetically) associated with Principled-JS was neuroticism, also hinting at the difficulties that being constantly sensitive to injustice can create. Relatedly, it was also surprising that Principled-JS did not have any kind of clear relationship with altruism, interpersonal trust, nor indeed with the Opportunistic JS factor (as tested in the model with an added genetic path from one factor to the other). Here we predicted substantial positive correlations for altruism and trust, and a negative correlation with opportunism, but found that all these correlations were very close to zero. Note however that our altruism scale (the Self-Report Altruism scale^48^) had a low Cronbach’s alpha of 0.48 in our sample, so our findings should be interpreted with this in mind. Nevertheless, it is consistent with previous findings that emotional empathy (as contrasted with cognitive empathy) is unrelated to a composite of Observer and Beneficiary sensitivity^61^, which are the highest loading facets on the Principled-JS factor. Together, this suggests that having an empathic and altruistic disposition may express itself as low Opportunistic-JS (i.e., lower victim-sensitivity *and* higher sensitivity to other injustices), rather than simply as high sensitivity to all injustice.

### Limitations and future directions

There are several limitations that should be considered when drawing conclusions from this study. Firstly, we only use self-report measures, which are known to be susceptible to certain errors and biases^62^. Of particular relevance to our study is social desirability bias^63,64^: It could very well be that certain responses to the JS scale and also to some of the other scales we use (e.g. altruism and social dominance orientation) could be more socially desirable than others, and that observed variances in these measures, and covariances between them, could be influenced by individual differences in the bias to respond in a socially desirable manner. Future work could investigate whether such influences indeed exist for our measures, and correct for them if they do. And, while the JS scale has indeed been validated as a predictor of certain relevant behavioral measures from e.g. economic games^65,66^, it could also be fruitful to investigate how principled and opportunistic justice-motivations relate to actual behaviors, such as reactions to experienced injustice, or behaviors reflecting empathy and altruism (or lack thereof).

Secondly, as this is a twin study, our conclusions regarding genetic and shared-environmental variances and correlations are subject to the assumptions and limitations that are inherent to twin designs. In particular, heritability estimates rely on the assumption that when correlations between monozygotic twins are higher than those for dizygotes, this is due to their higher genetic similarity, rather than e.g., systematic differences in how MZ and DZ twin pairs are influenced by the environment. For a more complete introduction to the logic and assumptions of twin designs, see e.g., Neale & Cardon^50^. As the field of behavioral genetics has seen rapid progress in recent years, it would be informative to study the heritability of justice sensitivity with approaches that make different assumptions than those of twin studies, such as other kinds of family designs, or designs utilizing measured genes.

Thirdly, the generalizability of our results is limited, since our sample consisted of twin pairs aged between 60 and 65 years, mostly born and raised in Norway. Our results might then not generalize to younger cohorts or to other cultures. Our findings on the heritability of JS do align with those from a Chinese twin sample with an age range from 18 to 25, however^40^. Still, it would be beneficial to broaden generalizability further, by investigating additional cultures and age-cohorts.

Fourthly, we use the short form of the JS scale. While this scale has shown impressive psychometric properties for its length^22^, our analyses would have benefitted from using the full 40-item JS scale, which is more reliable and valid.

An interesting avenue for future study, in addition to those suggested above as checks on our limitations, could be to investigate how Opportunistic-JS relates to the “dark triad” of personality traits^67^ (i.e., Machiavellianism, narcissism, and psychopathy). Our findings that it correlates negatively with agreeableness, altruism, and trust, and positively with SDO, are suggestive in this regard.

## Conclusion

Our study suggests that responses to the Justice Sensitivity scale can reflect both principled and opportunistic motivations. A principled motivation to preserve justice for its own sake leads to condemnation of all injustice, regardless of the perspective from which it is perceived. An opportunistic motivation to use justice in service of other goals rather leads to a more selective pattern of justice sensitivity; namely an *increased* sensitivity to injustices by which one is victimized, combined with a *decreased* tendency to feel shame and guilt when passively benefitting from, or actively perpetrating, injustice towards others. Both the principled justice sensitivity factor and, particularly so, the opportunistic factor in our model were found to highly heritable, with no significant proportion of variance attributable to the shared environment.

We found that measures of self-sacrificing and cooperative strategies for the greater common good were most strongly related to low moral opportunism, rather than to principled moral impartiality. This suggests that the absence of exploitative opportunism, rather than the presence of principled consistency, may form the core of moral character.

Our study highlights the complex relationship between justice sensitivity and morality. Moral rules generally have an in-built impartiality, such that they sometimes are beneficial to us and at other times are a hindrance. A principled person experiences motivations in proportion to what the situation demands in moral terms, regardless of who stands to benefit. The moral opportunist, on the other hand, has exaggerated or attenuated motivations, depending on what best serves their selfish interests in the current context, possibly without even being aware of it. For most people most of the time, responses to injustice will be compromises between both these kinds of motivations.

## Data Availability

Our data protection agreement does not allow us to share individual-level data from these studies to third parties.

## Code availability

Scripts of the statistical analyses are available upon request.
